# Acoustic Emission-Based Condition Monitoring and Remaining Useful Life Prediction of Hydraulic Cylinder Rod Seals

**DOI:** 10.3390/s21186012

**Published:** 2021-09-08

**Authors:** Jørgen F. Pedersen, Rune Schlanbusch, Thomas J. J. Meyer, Leo W. Caspers, Vignesh V. Shanbhag

**Affiliations:** 1 Department of Engineering Sciences, University of Agder, Jon Lilletuns vei, 4879 Grimstad, Norway; jorgenfp@uia.no; 2 Norwegian Research Centre, Technology Department, Jon Lilletuns vei 9 H, 3. et, 4879 Grimstad, Norway; rusc@norceresearch.no (R.S.); thme@norceresearch.no (T.J.J.M.); 3 Bosch Rexroth B.V., Kruisbroeksestraat 1, 5281 RV Boxtel, The Netherlands; leo.caspers@boschrexroth.nl

**Keywords:** hydraulic cylinder, acoustic emission, piston rod seal, root mean square, remaining useful life

## Abstract

The foremost reason for unscheduled maintenance of hydraulic cylinders in industry is caused by wear of the hydraulic seals. Therefore, condition monitoring and subsequent estimation of remaining useful life (RUL) methods are highly sought after by the maintenance professionals. This study aimed at investigating the use of acoustic emission (AE) sensors to identify the early stages of external leakage initiation in hydraulic cylinders through run to failure studies (RTF) in a test rig. In this study, the impact of sensor location and rod speeds on the AE signal were investigated using both time- and frequency-based features. Furthermore, a frequency domain analysis was conducted to investigate the power spectral density (PSD) of the AE signal. An accelerated leakage initiation process was performed by creating longitudinal scratches on the piston rod. In addition, the effect on the AE signal from pausing the test rig for a prolonged duration during the RTF tests was investigated. From the extracted features of the AE signal, the root mean square (RMS) feature was observed to be a potent condition indicator (CI) to understand the leakage initiation. In this study, the AE signal showed a large drop in the RMS value caused by the pause in the RTF test operations. However, the RMS value at leakage initiation is seen to be a promising CI because it appears to be linearly scalable to operational conditions such as pressure and speed, with good accuracy, for predicting the leakage threshold.

## 1. Introduction

Hydraulic cylinders are linear actuators that exert a linear actuating force with precise positioning and are used in a multitude of industry applications, such as bulk loading and handling systems, oil drilling equipment, compensating systems, and wireline tensioning systems. To contain the pressurized fluid inside the cylinder, a piston rod sealing system is used. In most cases, fluid leakage occurs in hydraulic cylinders because, over time, the piston rod seals lose their required sealing effect due to material degradation and mechanical wear processes. Additionally, seal wear occurs due to contaminants such as abrasive particles that are present in the fluid, excessive loads or pressures, or non-concentric rod and cylinder bores [1]. Furthermore, seal deterioration is accelerated by incompatible types of hydraulic fluid or high fluid temperatures. Because the seals are concealed within the system, the inspection of the seals cannot be made without at least partial disassembly of the cylinder [2,3]. Therefore, for a more practical solution to monitor the health of seals at regular time intervals, a condition monitoring (CM) strategy is needed. 

The total operating costs due to ordinary maintenance, excluding well maintenance, in the oil and gas (O&G) industry for 2021 are estimated to be NOK 17.5 billion (approximately USD 2 billion/EUR 1.7 billion) [4]. Therefore, even a minor reduction in maintenance costs can be of significant interest to the O&G industry. Condition-based maintenance (CBM) is one of the most cost-effective strategies to prevent the downtime of equipment and increase the productivity in the O&G industry [5]. Furthermore, the consequences posed by external leakage in hydraulic cylinders to human and environmental factors are considerable. Consequences such as increased chance of injury due to fluid spilling, risk of health problems, and risk of environmental contamination are consequences that outweigh the concern of cost. Thus, it is important to minimize the leakage, preferably avoiding it completely. Hydraulic cylinders are widely used in the O&G industry and thus the broadening of research on CBM for hydraulic cylinders is of significant interest to the O&G industry and for any other industries that depend on hydraulic cylinder operations where downtime is related to large costs.

Several papers have been published on possible methods used to diagnose leakage or monitor seal wear in hydraulic cylinders. An extensive review on the various methods found in the scientific literature was published by Shanbhag et al. [2]. Signal sources proposed for monitoring the degradation of seals in hydraulic cylinders are mainly pressure, vibration, and acoustic emissions (AE). The use of pressure sensors to diagnose leakage is the most common method due to its low cost. In the works of An and Sepehri [6,7], an extended Kalman filter (EKF) was applied as a state estimator for the chamber pressures. The residual error of the estimated states to the measured states was analyzed to detect the occurrence of leakage. Experimental tests were conducted by simulating internal and external leakage via an auxiliary hydraulic line in parallel to the hydraulic cylinder. It was shown that the residual error can be used to identify leakage at an early stage, with the magnitude of the residual error increasing proportionally to the increase in leakage. Furthermore by applying sequential analysis, the internal leakage rate was quantified between 0.6137 and 1.3221 L/min. Goharrizi and Sepehri [8,9] analyzed the pressure signal by applying a wavelet transform (WT)-Daubechies 8 (db8) wavelet to detect the internal leakage in hydraulic cylinders caused by seal wear. The investigation was based on the fact that internal leakage increases the damping coefficient of the hydraulic actuator, which intensifies as the leakage increases. The results show a change in the transient response of the pressure signal. Experimental tests showed that the RMS values of the level two and level one detail wavelet coefficients indicated a decrease in the presence of internal leakage related to the severity of the leakage, with the level 2 detail wavelet coefficient (d2) showing the highest sensitivity to the leakage condition. Eighteen tests with normal operating conditions, in addition to small (0.124 L/min on average) and medium (0.808 L/min on average) leakages, were conducted for repeatability and a threshold for internal leakage was set as the minimum RMS value for d2 of the normal operating conditions. By defining a threshold level, 80% of the small leakage tests were able to be identified. Furthermore, the external leakage was found to be similarly sensitive to the level four approximate wavelet coefficient of the pressure signal. Thus, the internal and external leakages could be isolated when both conditions occurred simultaneously. Results from nine separate experimental tests utilizing both internal and external leakage conditions at 0.36 and 0.26 L/min, respectively, showed the occurrence of both leakage conditions was able to be detected in 90% of the tests. In another work by Goharrizi and Sepehri [10], the Hilbert Huang transform (HHT) was applied to the pressure signal to investigate internal leakage in hydraulic cylinders. Using this method, it was possible to detect internal leakage as low as 0.124 L/min for a periodic step position reference input signal and 0.230 L/min for a pseudo-random position reference input signal to the actuator control valve. Furthermore, the sensitivity of the approach was shown to increase with the severity of the leakage. The benefit of the HHT method is that a mother wavelet does not have to be chosen a priori; however, it is more computationally expensive than the WT approach. Zhao et al. [11] used wavelet packet analysis to investigate pressure signals along with the displacement signal to diagnose and predict early leakage in hydraulic cylinders. Experimental tests were conducted utilizing different orifices between the inlet and outlet pressure lines in parallel to the cylinder to simulate leakage. The wavelet packet energy variance was found to have the highest sensitivity to the early leakage in hydraulic cylinders. In other work, Zhao and Wang [12] used a fiber Bragg grating sensor to measure the contact strain on the piston seals during operation to investigate the wear features in hydraulic cylinder piston seals. Experimental tests were conducted with three piston seal conditions: unworn, semi-worn, and worn piston seals. From the time domain feature analysis, the margin index and kurtosis were observed to have the strongest sensitivity for the degree of seal wear.

Tan et al. [13,14] and Yunbo et al. [15] investigated the use of vibration sensors to diagnose faults in water hydraulic actuators. A linear actuator was tested with unworn and worn piston and rod seals to diagnose both external and internal leakage. It was observed that, with an increase in piston seal wear, a decreasing trend in vibration energy occurred. This was attributed to the increase in leakage caused by the increase in wear; the fluid then acts as a liquid seal and makes the stroke smoother, resulting in a lower vibration signal. The authors also investigated rod seal wear, where the average stroke time was used as a measure of wear. In this work, it was observed that a decrease in average stroke time occurred with increasing rod seal wear. According to the authors, this trend was observed because the unworn seal has a higher frictional force between the rod and the seal, which results in an increase in the force required to move the piston.

An area of current interest in research literature is the use of AE sensors for CM data from hydraulic cylinders. The use of AE for this purpose is less established and research in this field saw scarce effort until the last 15 years. The use of AE sensors for CM possesses the benefit of having a very high frequency range; as a result, it is largely unaffected by the machine noise. Chen et al. [16] used AE to investigate the relationship between AE signals and the internal leakage rate (lower than 1.0 L/min) in a water hydraulic cylinder. Piston seal failure was simulated by filing small channels in the piston seal. The internal leakage rate and AE signal energy measured in either the time or frequency domain was found to be very strongly linearly correlated with the fault severity. Shanbhag et al. [17,18] provided the most recent research on the diagnostics of hydraulic cylinders using AE. Experimental studies were conducted using AE on a hydraulic cylinder using a water-glycol-based fluid in a test rig. The time and frequency domain features for different degrees of seal and rod wear were investigated. It was observed that the mean frequency and median frequency could be applied to detect both seal and rod degradation in the test rig over a large range of working pressures. Furthermore, the influence of rod speeds was investigated for both the unworn and worn seal conditions, where rod speeds of 50 and 100 mm/s were tested, together with 10, 20, 30, and 40 bar working pressures. The bandpass filtered signal between 50 and 100 kHz and the unfiltered signals were analyzed through time and frequency domain features. In this study, the AE RMS, peak, and mean frequency features of the bandpass filtered signal could separate the seal conditions for both speed conditions with good separability. 

AE can indicate wear initiation at microscopic levels, and can be used to simultaneously monitor multiple parts using a single sensor, such as both piston rod, bearing, and piston rod seal wear [2]. Therefore, the focus of this study was to understand the initial stages of seal wear and leakage initiation using AE, and to predict and estimate the remaining useful life (RUL) using the AE features. The use of AE features has been found to be promising in distinguishing various degrees of piston rod seal and piston rod wear, and has shown good separability over a range of different working pressures and rod speeds. However, there is a lack of studies investigating the seal degradation over a period of continuous cylinder operation and thus the initial stages of external leakage in hydraulic piston rod seals. Therefore, in this work we investigated the external leakage initiation by performing a set of run to failure (RTF) experimental tests using AE sensing. Furthermore, all previous work on applying AE sensors to hydraulic cylinders was performed by attaching the AE sensor directly to the piston rod. In fielded systems, this may not be feasible for some applications due to several reasons, e.g., if the rod fully retracts into the cylinder. Therefore, this work also investigated the applicability of different sensor locations.

## 2. Materials and Methods

**Figure 1:**
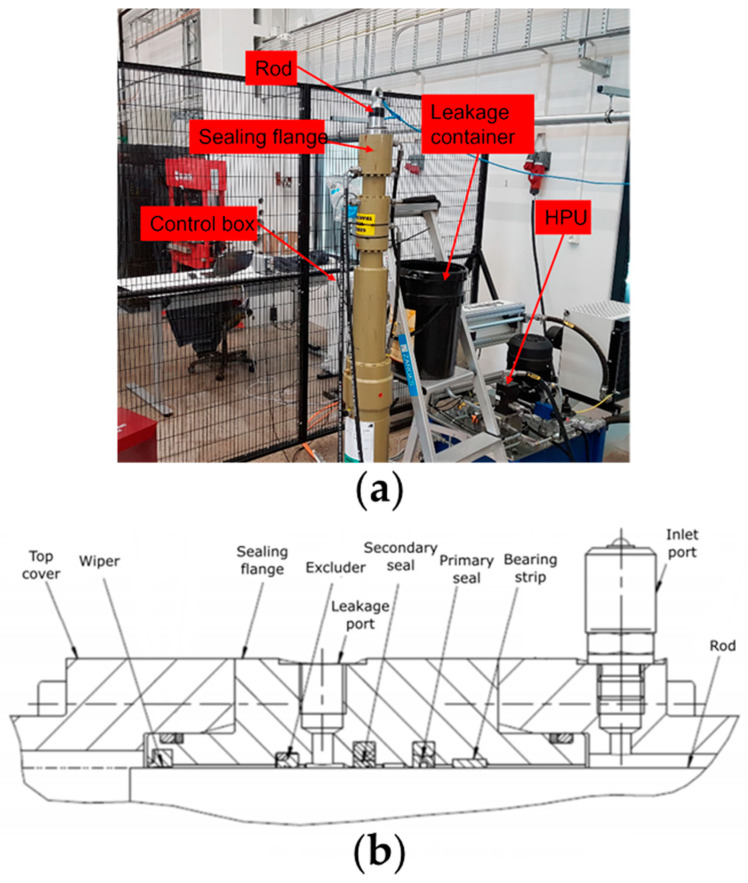
(**a**) Overview of the test rig; (**b**) section view of sealing flange.

The test rig used in this research consisted of a hydraulic power unit (HPU) that supplies pressure to a pressure chamber located inside the cylinder head, controlled via a pressure control valve as seen in Figure 1a. A servomotor drives a ball screw spindle inside the lower part of the test rig, which is used to move the rod back and forth through the pressure chamber. The piston rod seals are placed in both ends of the pressure chamber flange sealing flanges. There are four different rod sealing elements in the sealing flanges: one primary and one secondary seal, one excluder, and one wiper. Additionally, one bearing strip is used to withstand possible side and bending loads. Only the primary and secondary seals retain the hydraulic fluid from the pressure chamber before reaching the leakage port. The test rig, designed and built by Bosch Rexroth BV, was built to closely replicate the normal operating conditions of a hydraulic linear actuator. Figure 1b shows a cross-section view of the sealing flange. From the leakage port, a hydraulic fluid hose with a length of 50 cm is connected to lead fluid leakage out of the cylinder head into an external container. The test rig is operated by a dedicated PC that runs the software controlling the servomotor for the ball screw spindle. The piston follows a trapezoidal input signal, and, in the software, the stroke length, rod speed, and acceleration can be adjusted. Consequently, for all tests, the acceleration was set to twice the value of the velocity. Hydraulic test rig details used in this study are summarized in Table 1. 

**Figure 2:**
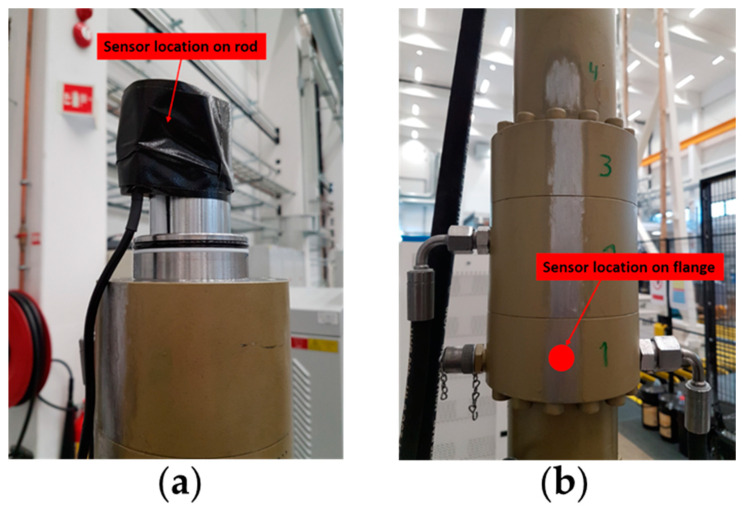
Sensor locations: (**a**) on rod (sensor mounted and secured with tape); (**b**) sensor location on the upper flange.

**Figure 3:**
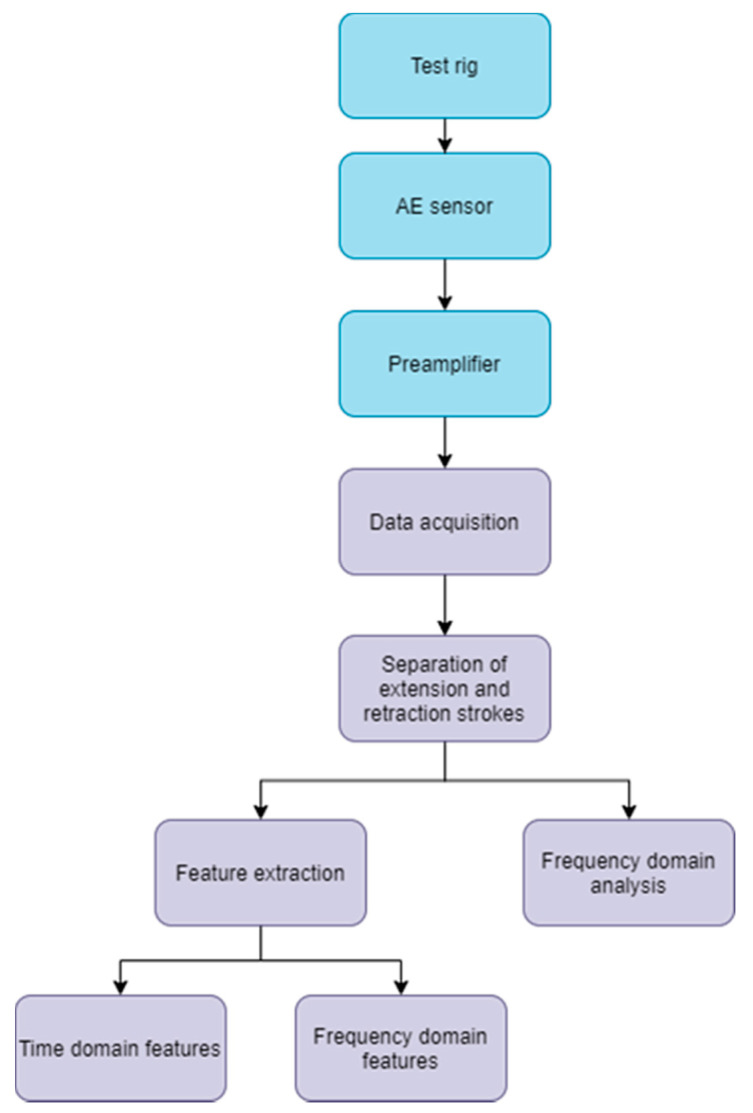
Signal path for AE data acquisition and analysis.

The AE sensor was mounted on the test rig using an adhesive bond together with tape to prevent the sensor from falling and getting damaged if the adhesive bond is broken. The adhesive bond secures a good signal path. Figure 2 shows the different sensor locations used in this study. Two different sensor locations were used for the RTF tests: (a) directly on the piston rod, and (b) on the upper flange section. The sensor position on the upper flange section was chosen because the sampled signal was strongest at this location. The sensor is a general-purpose narrow-band resonant sensor. The frequency operating range of the sensor is 50–400 kHz and the resonant frequency is 150 kHz, which provided a good sensitivity and signal to noise ratio. The AE sensor was connected to a preamplifier with a selected gain of 40 dB (0/2/4-Switch selectable gain single-ended and differential preamplifier, Supplier: Physical Acoustics). The preamplifier was connected to the AE data acquisition system (AMSY-6, Supplier: Vallen systeme) further connected to an external PC through the USB port. The software accompanying the AMSY-6 system, Vallen AE suite, was used to initiate data acquisition. Figure 3 shows a flowchart of the signal path used for AE data acquisition and analysis. To verify the correct mounting of the AE sensor, the Hsu-Nielsen pencil lead break test was performed. The pencil lead break test consists of breaking a 0.5 mm diameter pencil lead on the test rig surface near the mounted sensor.

To determine the suitable AE features that can be used to monitor the external leakage in the test rig, five RTF tests were conducted. The time of leakage initiation was defined as the time when the first drop of hydraulic fluid could be observed from the leakage hose. The tests were performed until leakage initiation occurred and then continued for a few hours after. Time to leakage initiation was accelerated by creating multiple longitudinal scratches on the rod to create microscopic leakage paths. Furthermore, the scratches created a metal build-up along the scratch edge that, to some degree, induced scarring on the seals. The sensor location and the rod speed condition were varied between tests to investigate the effect on the RTF test signal. In addition, for the third and fourth RTF tests, the test rig was paused overnight to observe the effect on the AE signal. Each test was sampled intermittently at 15 min intervals to save disk space and reduce the time required for data analysis. Each sample was acquired for approximately 90 s, rounding up or down to ensure the piston completed the full stroke cycle. Table 2 summarizes the process parameters for the experimental RTF tests.

**Figure 4:**
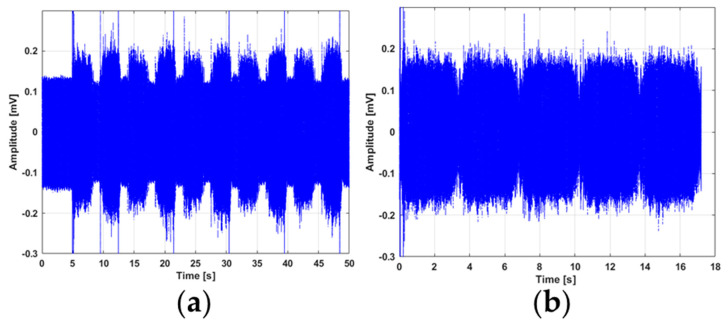
AE signal recorded from the experimental test rig, from the test at 100 bar and 50 mm/s rod: (**a**) original data; (**b**) pre-processed data (extraction stroke only).

The AE signal can be sampled in two different measurement modes: continuous mode and burst mode. In continuous mode, the continuous time stream of the AE signal is sampled, whereas in burst mode only the AE signal above a predefined threshold is sampled. All experimental tests conducted in this work were sampled in the continuous mode. The continuous AE signal, as seen in Figure 4a, shows five consecutive strokes. Each extension and retraction part of the signal can be clearly identified, in addition to the dwell time between. The signal thus shows a clear repetitive pattern corresponding to the motion of the piston rod, in addition to some clearly defined spikes, indicating that the time series is non-stationary. Because some of the extracted features are based on the fast Fourier transform (FFT), it was necessary to attempt to create a stationary series. For this purpose, the dwell time section between each stroke was removed from the time series. The major spikes occurring in the time series mainly occurred at the end of the retraction stroke. In addition, the extension and retraction strokes appear to be very similar in both variance and length. As a result, only the extension strokes were used for further data analysis. The pre-processed signal thus consisted of the extension strokes only, concatenated back-to-back, to establish a close-to-stationary process signal, as seen in Figure 4b.

A set of seven different statistical features were extracted from the pre-processed signal. The features are comprised of energy-based features, frequency-based features, and distribution shape-based features to provide a good overview of how the signal responds over time. Furthermore, the standard error was calculated for each of the features across the strokes used in the preprocessed data series.

In Table 3, *x*(*n*) is the discrete signal for *n =* 1, 2, 3, …, *N*, where *N* is the total number of data points in the series. *x_std_* and *x_m_* are the standard deviation and the mean of the discrete signal, respectively. *s*(*k*) is the spectrum for *k =* 1, 2, 3, …, *K*, where *K* is the total number of spectrum lines. *f_k_* is the frequency value of the *k*th spectrum line [19]. To compare the frequency domain of different test samples, the second extension stroke was extracted and used for the analysis. This limited the number of data points and saved time required for computation. Welch’s method was used to create the power spectral density (PSD) estimate. A first-order Kaiser window with 50% overlap was used to get a smaller error variance in the final estimate. The number of discrete Fourier transform points were set to 1000 to give a smooth plot line.

## 3. Results

### 3.1. AE Features

**Figure 5:**
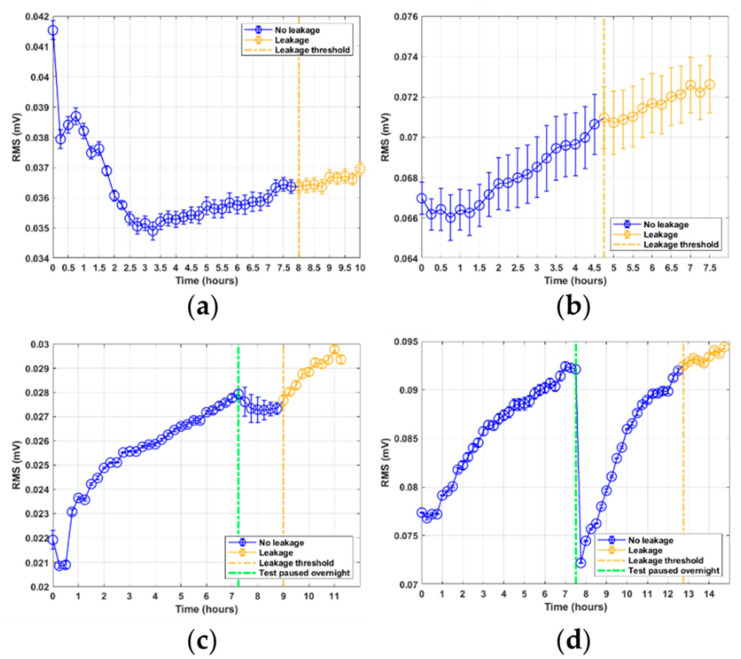
RMS feature results from RTF tests 1–4: (**a**) test 1; (**b**) test 2; (**c**) test 3; (**d**) test 4.

The RMS features shown in Figure 5a–d can be seen to have at least two distinct phases. There is an initial drop in signal energy, most notably seen in tests 1, 2, and 3. After this phase, there is a phase of steady increase in signal energy that can be seen in all four tests. For tests 3 and 4, there can also be seen a sudden drop in energy. This drop is known to correspond to the time where the test rig was paused overnight; this is marked in the plot by a green line. It should be noted that the fourth test has a noticeably larger drop, because on the third test, the test rig was switched on and kept running for a while before the first sample was obtained after the overnight pause. The rate of increase in the RMS levels after the pause is larger than the initial rate of increase for the first few hours. However, when the RMS values approach the previous levels from before the pause, the RMS rate of increase reduces, eventually continuing the trend that existed before the tests were paused. The average energy is higher for both tests conducted with the sensor mounted at the flange section. Furthermore, the average energy increases with an increase in rod speed. For the RTF test 1 in Figure 5a, there is an initial large decline in the RMS values, particularly between the first and second samples. Subsequently, there is a slight increase, before the decline continues until around three hours, after which it starts to increase again. This transient response of decreasing RMS levels is also replicated in tests 2–4. However, in this case the transient response ends after 30–45 min. For all tests, the RMS level continues to increase for the remaining time of the test. The standard error for the RMS feature is low and nearly constant for tests 1, 3, and 4. For test 2, however, the standard error is noticeably larger for all samples, although it remains close to constant in value.

**Figure 6:**
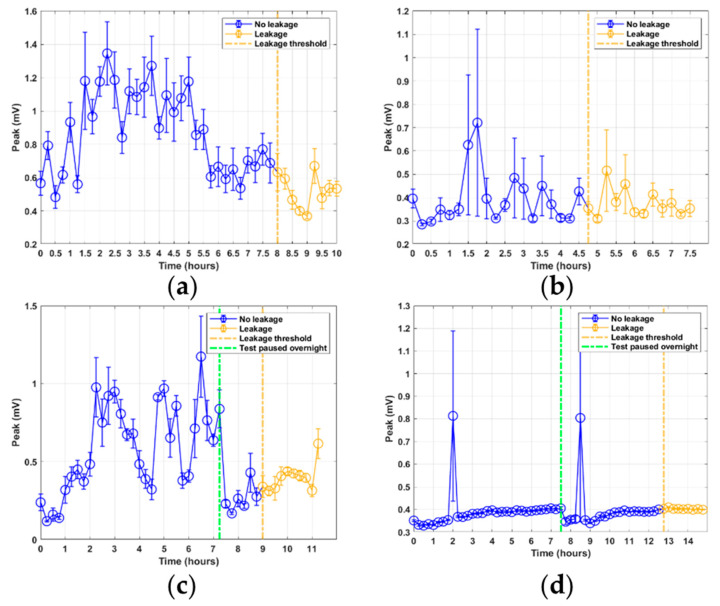
Peak feature results from RTF tests 1–4: (**a**) test 1; (**b**) test 2; (**c**) test 3; (**d**) test 4.

**Figure 7:**
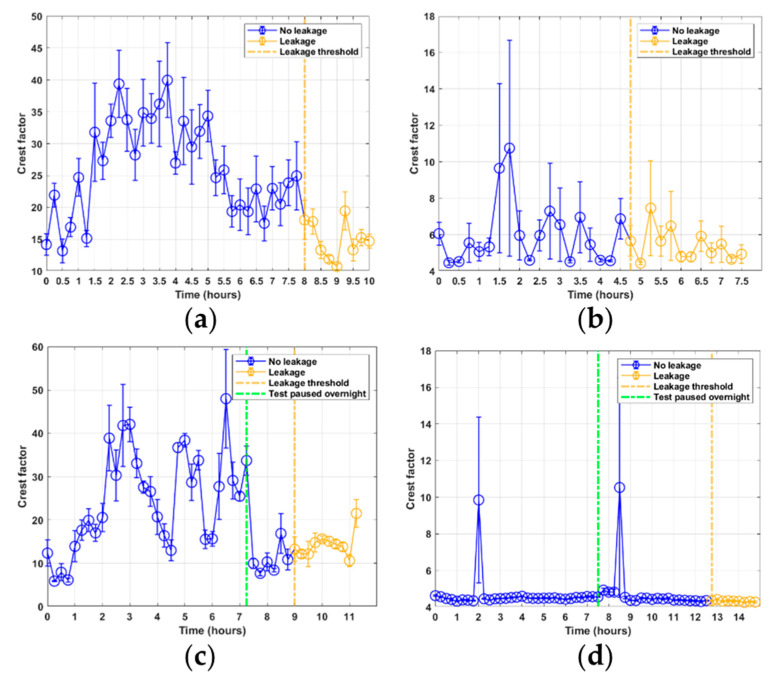
Crest factor feature results from RTF tests 1–4: (**a**) test 1; (**b**) test 2; (**c**) test 3; (**d**) test 4.

The peak feature shows that for most of the samples in the RTF tests 1–3, as seen in Figure 6a–c, peaks occur within the signal that are much higher in energy than the general RMS levels. These peaks can be attributed to AE burst events that occur during the sampling. The average energy level of the burst signals can be seen to be approximately at the same level for all tests. It is interesting that the RMS levels show a clear relationship between signal energy and the rod speed and sensor location, whereas the peak energy does not show a similar trend. Furthermore, the peak levels for RTF test 4 in Figure 6d show a lack of burst events occurring for large parts of the samples. Only two of the 60 samples in RTF test 4 can be seen to experience burst events. For the largest peaks, the standard error is also high, indicating that the peaks are largely individual. The crest factor in Figure 7a–d shows a very similar response to the peak feature, as may be expected due to their mathematical relationship.

**Figure 8:**
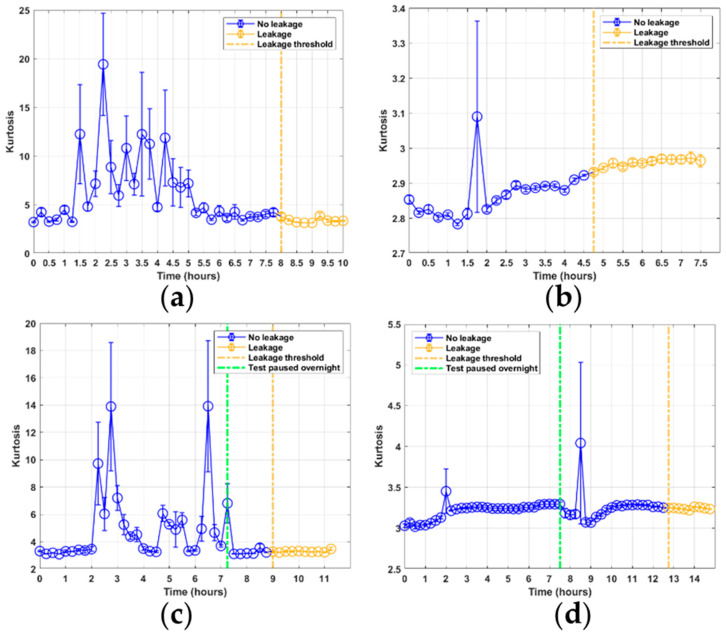
Kurtosis feature results from RTF tests 1–4: (**a**) test 1; (**b**) test 2; (**c**) test 3; (**d**) test 4.

**Figure 9:**
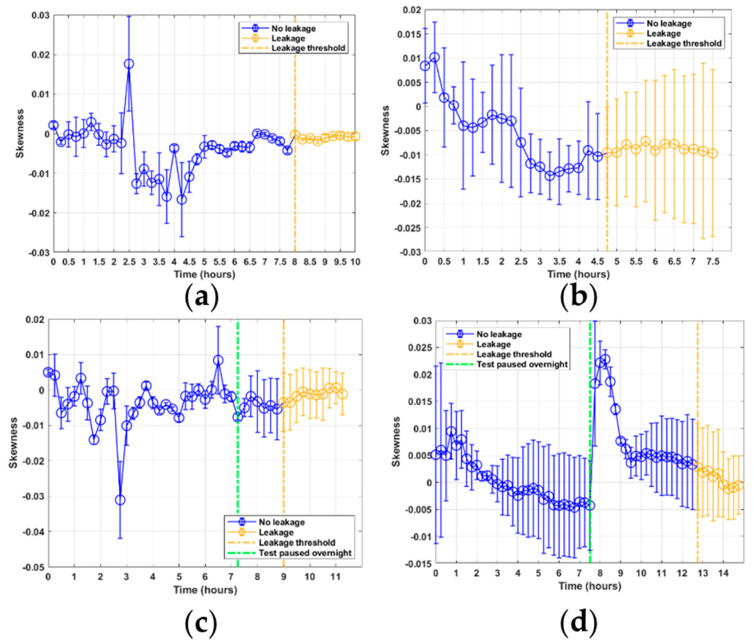
Skewness feature results from RTF tests 1–4: (**a**) test 1; (**b**) test 2; (**c**) test 3; (**d**) test 4.

The kurtosis features for tests 1 and 3, shown in Figure 8a,c shows some similarities in their response. For both these tests, the AE data was sampled with the sensor mounted on the piston rod. Both can be seen to lie steadily at kurtosis levels of approximately 4 before experiencing some large peaks several hours into the tests, which then appear to stabilize again towards the end of the tests. These responses can be related to the burst events occurring during each sample because the larger burst signals alter the shape of the distribution. For tests 2 and 4, in Figure 8b,d, the AE data were sampled with the sensor mounted on the flange section. Here, the kurtosis shows a different response, where peaks are only observed when the burst events occur, and otherwise steady levels are observed. The skewness levels for all tests shown in Figure 9 can be seen to show no significant trend that can be related to leakage or seal degradation. Furthermore, the skewness values do not exceed a ±0.5 skewness factor, meaning that each of the sample signals can be considered to be approximately symmetric ([20], p. 63).

**Figure 10:**
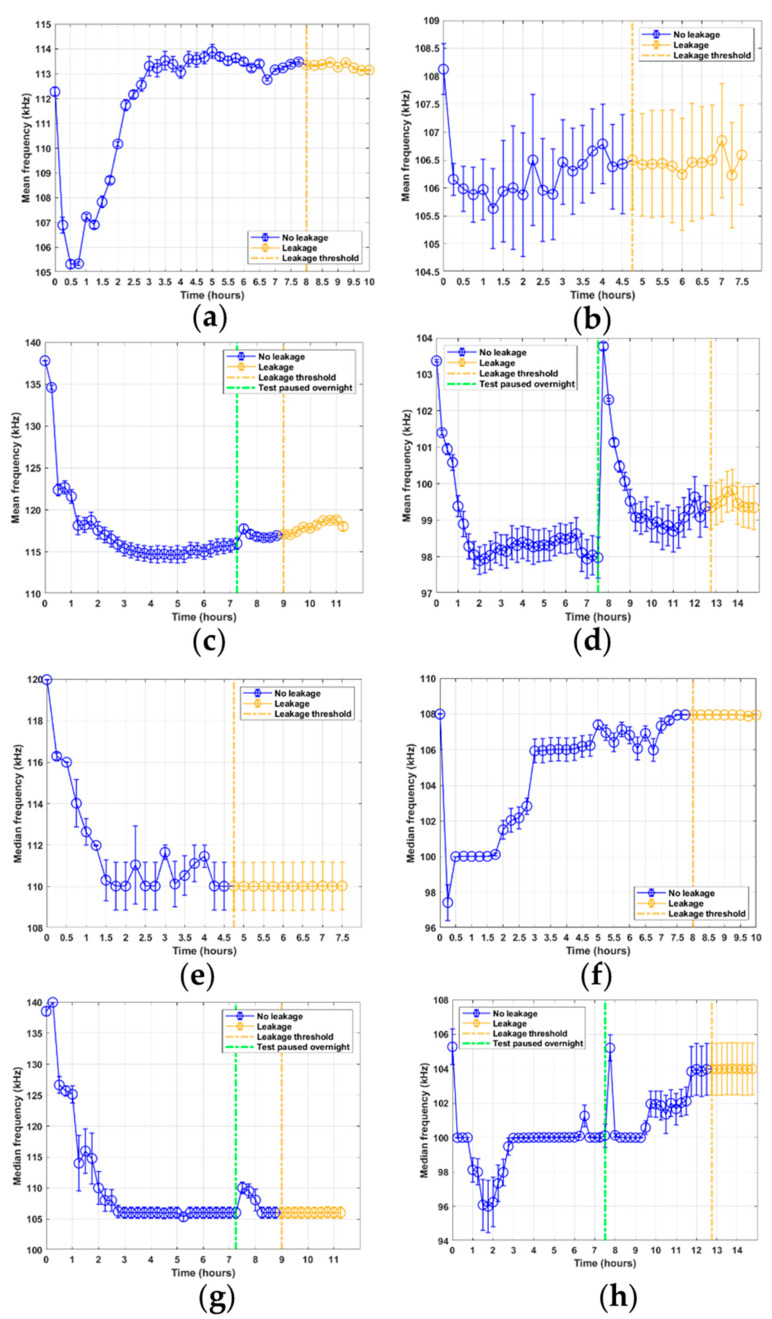
Mean and median frequency feature results from RTF tests 1–4: (**a**) mean frequency, test 1; (**b**) mean frequency, test 2; (**c**) mean frequency, test 3; (**d**) mean frequency, test 2; (**e**) median frequency, test 1; (**f**) median frequency, test 2; (**g**) median frequency, test 3; (**h**) median frequency, test 4.

The mean and median frequencies are shown in Figure 10. Both show an interesting time trend in the initial samples for all RTF tests, experiencing a drop in frequency between the initial sample and the next two to five samples. However, particularly for test 3 in Figure 10c,g, the frequency levels for the initial data points are significantly different from the latter data points. For test 3, the mean and median frequencies of the first two samples occur between 135 and 140 kHz, and are considerably higher than those for any other dataset. For test 4, the initial mean frequency occurs at 104 kHz, as seen in Figure 10d, before stabilizing at around 98 kHz. Then, after the overnight pause of the test rig, the process is repeated. Qualitatively, no other correlation can be seen between the different datasets for the mean and median frequencies.

Based on these results alone, it is hard to determine any deciding event in the features that might suggest the onset of leakage. There seems to be, at least for tests 1, 2, and 4, a small sudden upwards shift in the RMS value between one to three samples before the leakage was first observed. This small shift is, however, not unique to the observation of leakage, because similar shifts appear at other instances in time, as is especially seen in test 2. Additionally, the median frequency showed an increase where the leakage was first observed in test 4. However, the same problem occurred here because it was not an event that was replicated in the other tests. Thus, it cannot be concluded that these events in the feature signal are a deciding feature of leakage initiation alone. For use as CM data, based on visual inspection alone, only the RMS feature shows promising behavior in being correlated with the degradation of seals due to its steadily increasing trend.

### 3.2. Long-Duration RTF Test 5

**Figure 11:**
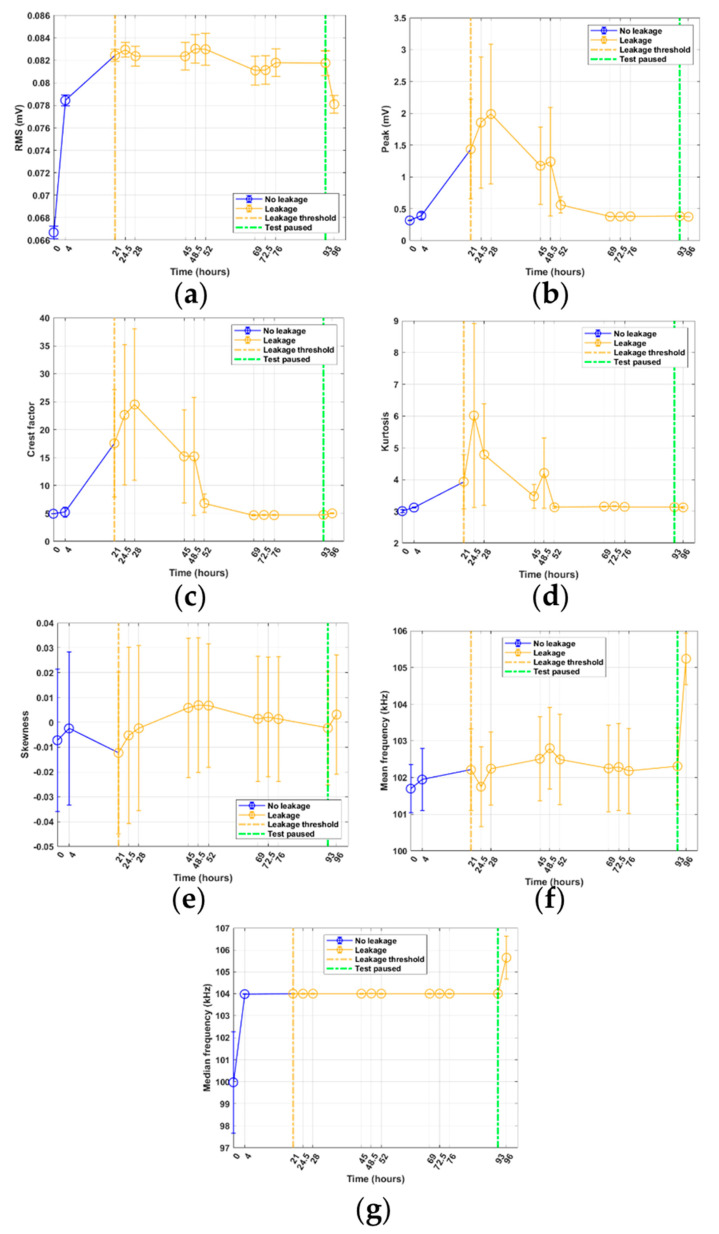
Feature results from RTF test 5: (**a**) RMS; (**b**) peak; (**c**) crest factor; (**d**) kurtosis; (**e**) skewness; (**f**) mean frequency; (**g**) median frequency.

The long-duration RTF test, known as RTF test 5, was initiated on a Monday at 12:30; thus, only two data samples were acquired that day at 12:30 and 16:00. At a time between 16:00 on day one and 09:00 on day two, leakage occurred; the leakage occurred at between 4 and 21 h of operation (Figure 11). After the initial leakage, the leakage remained at a steady rate for the remainder of the test duration, meaning no total seal failure occurred. Figure 11a shows the RMS plot for RTF test 5. The plot shows a similar response to RTF tests 1 to 4, with the increasing trend in signal energy after the first several hours. However, after a certain time, the signal energy settled and maintained an approximately constant level for the remaining duration of the test. The last data point in the plot shows a drop in signal energy similar to that of RTF tests 3 and 4 due to the pause in the test rig operation between the second to last and the last data sample.

### 3.3. Frequency Domain Analysis

**Figure 12:**
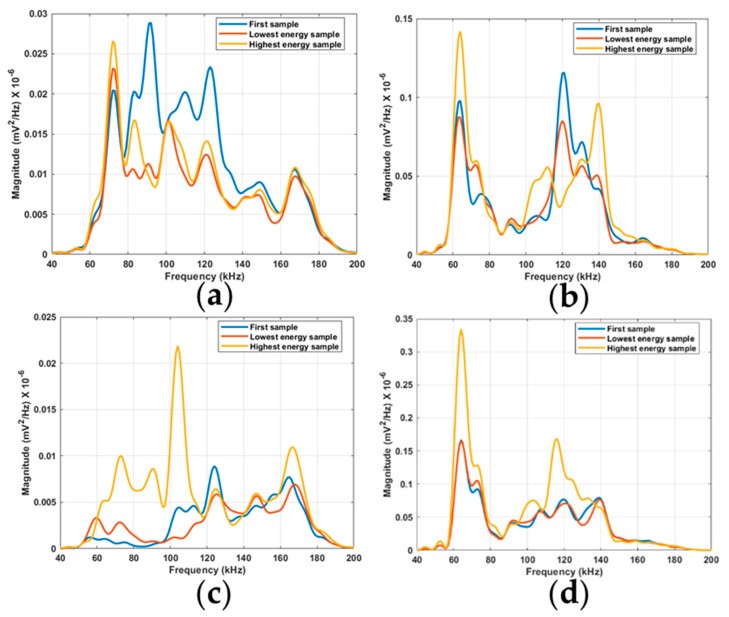
PSD response of RTF tests 1 to 4 for the first, the lowest energy, and the highest energy samples: (**a**) test 1; (**b**) test 2; (**c**) test 3; (**d**) test 4.

To investigate whether bandpass filtering may be applied to the RTF test results to correlate the response more closely to the seal degradation, the test data was analyzed in the frequency domain. It was seen that, for all tests, the frequency responses all showed significant energy in the range between 40 and 200 kHz; thus, all plots are shown only in this frequency range for clarity. This frequency band corresponds well to the frequency characteristics of the AE sensor that were used with the resonant frequency at 150 kHz. To investigate the initial trend of decreasing energy seen in the RMS plots, the first sample of each RTF test is plotted together with the initial corresponding lowest RMS energy sample (blue and orange lines in Figure 12). Furthermore, the highest RMS value sample is plotted to compare the frequency response of the steady increase in signal energy (orange and yellow lines in Figure 12). First, a preliminary inspection of the frequency responses was conducted by comparing the overall response of the samples that were investigated. For tests 2 and 4, there appear to be similar responses across the frequency band between 40 and 200 kHz, both experiencing initial peaks at 64 kHz, then a dip occurring between 80 and 100 kHz, before higher activity occurs again between approximately 100 and 150 kHz. Both tests 2 and 4 had the sensor mounted on the flange section. For tests 1 and 3, which both had the sensor located directly on the rod, the similarities are much less prominent. The peak occurring at 72 kHz for test 1 can also be seen in test 3, but to a much lower extent. In addition, similar peaks between tests 1 and 3 can be seen at 124, 148, and 167 kHz. For all tests, regardless of the sensor location, peaks at around 120 kHz can be recognized.

The initial drop in the RMS signal energy seen in Figure 5 was investigated. The largest initial drop was recorded for test 1. Then, for the remaining tests, the initial drop was much lower, but a noticeable trend can still be seen in all tests. The frequency band between 77 and 160 kHz has an increase in energy compared to the lowest energy sample. For the frequency response of test 2, it is mainly the frequency band between 117 and 135 kHz that shows higher energy in the first sample. For test 3, the frequency response shows higher energy for the first sample, mainly in the frequency band between 117 and 130 kHz. For test 4, there is barely any distinction in signal energy between the first sample and the initial minimum energy sample. This is expected due to the low response of this initial drop in signal energy already implied by the RMS feature. For the steady increase in signal energy for test 1, the response appears to be very similar throughout the frequency range between 40 and 200 kHz. The only large difference occurs between 80 and 90 kHz, where the highest energy sample clearly has larger energy. For tests 2 and 4, the same frequency band between 60 and 80 kHz can be seen to show a large difference in signal energy between the lowest energy sample and the highest. For higher frequencies, the signal energy from the highest energy samples appears to be concentrated at 117–140 kHz for tests 2 and 4, respectively. For test 3, the entire frequency band between 60 and 120 kHz shows an increase in energy. Furthermore, an increase can be seen with the peak occurring at 167 kHz. Most promising is the frequency band for bandpass filtering, which occurs between 60 and 80 kHz for tests 2 and 4, indicative of results similar to those expected based on the RMS energy results. This frequency band also shows the expected response for RTF tests 1 and 3, although to a lesser degree. Based on this, the signal was bandpass filtered between 60 and 80 kHz for all tests to investigate the results.

**Figure 13:**
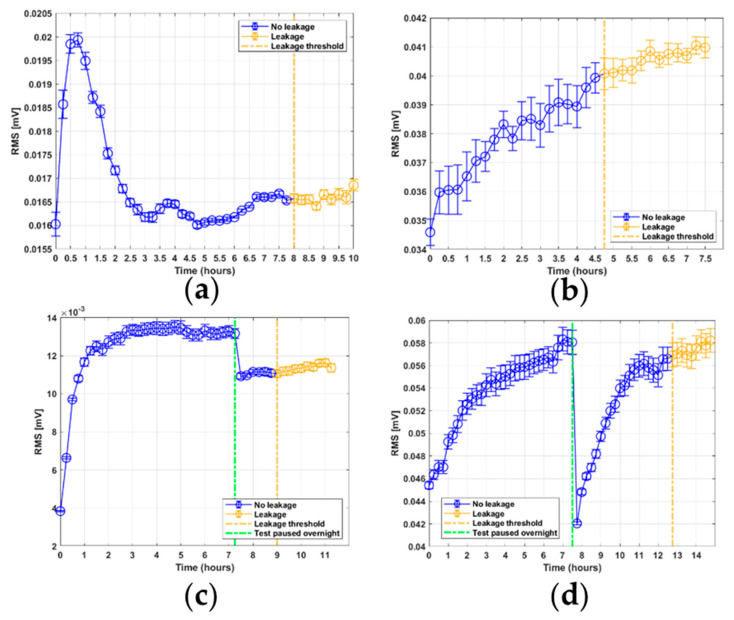
RMS feature from RTF tests, bandpass filtered between 60 and 80 kHz: (**a**) test 1; (**b**) test 2; (**c**) test 3; (**d**) test 4.

Figure 13 shows the RMS feature for the bandpass filtered signal. For the tests conducted with the sensor mounted on the flange section, tests 2 and 4, the response closely follows the response previously seen in the unfiltered signal. However, the initial decreasing trend is not present in this frequency band. However, the trend with the large drop in RMS levels after a pause of the continuous operation is still present. For the tests conducted with the sensor mounted on the rod, tests 1 and 3, the response can be seen to show a quite different trend to the unfiltered RMS signal. Both plots have a large transient response before settling at a more constant level, and there appears to be no indication of leakage initiation. Based on these results, for use as CM data, bandpass filtering is applicable to gain a better signal correlated to leakage for tests that have the sensor mounted on the flange section due to the removal of the initial decreasing trend.

### 3.4. Dependence on Test Conditions for Leakage Initiation Threshold

**Figure 14:**
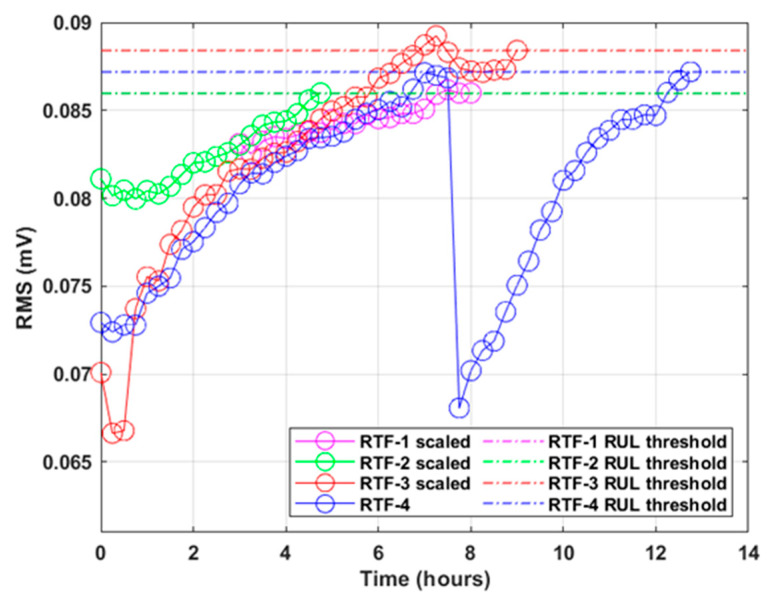
RMS data from all RTF tests scaled by location and speed factors.

The dependence of the test conditions on the RMS feature was tested by first finding the RMS levels at the time of leakage initiation for each RTF test. The ratio between the RMS value for the tests conducted with the sensor mounted on the flange, to the tests conducted with sensor mounted on the rod, was then calculated for both speed conditions. The equations used to calculate the scale are shown in Table 4, where *L* is the sample in which leakage was first observed and *x_rms_* is the RMS values for the RTF test. Ratios showing a similar value provide evidence that the RUL threshold can be calculated based on the remaining tests. Similarly, the ratio between the 15 and 25 mm/s rod speed conditions was calculated. Table 5 shows the calculated speed and location scale factors. Both the speed and the location ratios (scale factors) are very close in value, with a standard deviation of 0.0246 for the location scale and 0.0134 for the speed scale. Figure 14 compares the scaled RMS series from all tests together with the RUL threshold. These calculated scaling factors can then be applied to scale the currently sampled RMS data, and scale it to fit any other test data RUL threshold, based only on the current test speed and sensor location conditions.

## 4. Discussion

From the set of AE features for all experimental tests that were conducted, the RMS feature stands out as being best able to identify the specific conditions. A trend clearly seen in the RMS feature for all RTF tests was the initial drop in RMS levels, most notably seen in the results from test 1. It is important to note that for test 1, all seals, and primary, secondary, excluder, wiper, and bearing elements, were changed to new unworn seals. In contrast, for the remaining tests, only the main and secondary seals were changed. As a result, it is believed that this initial large drop for test 1 was caused by a wear-in or run-in period of the seals. A run-in period is a period of initial high contact stress, and thus high friction force, as the new and unworn seal conforms to the sliding of the rod. This run-in period would cause a rapid decrease in contact stress and friction force, which would result in a correspondingly rapid decrease in AE energy, as seen by the RMS feature. These results strongly indicate that the seals were highly worn in the regions where the largest contact stresses occur as a result of the assembly process. This wear pattern is recognized in the literature for wear processes in polytetrafluoroethylene (PTFE) seals and is attributed to the nonlinear elasto-viscoplastic material properties of the sealing material, which cause the seal wear to be dependent on the mounting process. Furthermore, a period of run-in for newly assembled parts is a widespread phenomenon seen in most mechanical processes. A similar theory is examined in other research that investigates the relationship between friction and wear [14,21,22].

Shanbhag et al. [23] performed a similar RTF test with a similar experimental setup. This RTF test was run continuously for approximately 17 h using all unworn seals. However, in their experiment, the authors did not experience a similar initial large decrease in RMS energy. Shanbhag et al. [23] utilized a bandpass filter from 50 to 100 kHz in their experimental tests, because the authors previously identified this frequency band to correspond to the seal wear process. It should be noted that, in this previous study, the type of AE sensor, and its placement directly on the rod, was the same as that used in the RTF tests 1 and 3 in the present research. From the frequency domain analysis, the frequency band between 60 and 80 kHz was identified as the frequency band in which the seal wear is most prominent for the experiments conducted in this study. Thus, the identified frequency bands for seal wear of both the experiments of Shanbhag et al. [23] and the current experiments in this paper occur in an overlapping frequency region. In Figure 13, the bandpass filtered RMS series from all tests show that the initial decreasing trend is removed from the RMS series. This implies that the run-in period could not be identified in the experiments of Shanbhag et al. [23] due to the bandpass filter. However, because the frequency band in the experiments of Shanbhag et al. [23] increased to 100 kHz, whereas in the current research it was only shown up to 80 kHz, it cannot be excluded that the run-in period may be identified if the same bandpass filter were applied. However, it is a strong indication that the run-in period was of a higher frequency and thus was not captured in the experiments of Shanbhag et al. [23].

Another theory is provided by the fact that the trend can be seen to correlate well with another phenomenon that occurred during testing, namely, the initial rotation of the rod around its central axis during the continuous operation of the test rig. This behavior is attributed to the machining process of the rod itself and the manner in which it is mounted on the spindle drive. The rod is machined via a turning process and, despite the extremely fine surface finish, small radial grooves caused by the turning process exist on the surface. These radial grooves act as an extremely fine screw, slowly rotating the rod while under continuous operation. The first evidence of this was seen after an initial trial run of the test in which the test rig was run overnight. By morning, the rod had rotated multiple rotations and caused the sensor cable to coil around the rod. Subsequently, the issue was fixed by mounting an eyebolt on top of the rod with a rope attached offset from the rod’s central axis and to a fixed point on the wall next to the test rig. This provided a counter torque to the rotation and subsequently stopped the rotation as the rope became tight. However, to prevent overtightening of the rope and hindering of the rod’s axial movement, the rope requires initial slack at the beginning of each test. The rod rotation was then used to tighten the rope to its optimal tightness. Thus, some degree of rod rotation was seen in the starting phase of the RTF tests. It can be noted that, for test 1, this period of rod rotation was longer than for the remainder of the tests, at approximately three hours. As confidence increased regarding the rope’s initial tightness, the remaining tests were undertaken with a tighter rope. Thus, it cannot be ruled out that this phenomenon is correlated with the initial large drop seen in the AE signal RMS feature.

The use of the RMS feature for CM data appears to be promising due to its steadily increasing values, and because the leakage thresholds showed similar values across test conditions. However, the cause of the steady increase is not conclusive based on these results alone. In the previous section, the theory of a run-in period was discussed. This theory implies that the AE RMS feature is highly correlated with the stress and friction of the seal contact and, hence, the seal wear processes. However, for the steadily increasing trend after the initial decreasing trend, a different process is proposed to be the cause. In the RMS series for RTF test 4 in Figure 5d, the RMS signal drops to below its initial value after an overnight pause of the test rig. This provides evidence that the AE RMS feature is not correlated with the seal wear after the end of the initial decreasing trend, because then the RMS levels would have continued at the same levels as before the pause. In the RTF test 5 results, it was seen that the increase in RMS level would eventually stop, then remain at a continuous level for the remining time of the test. In a paper by Chen et al. [24], a similar response was found when investigating the internal leakage of a hydraulic cylinder using AE data. Here, the leakage paths were induced by filling small grooves in the piston seal to allow the fluid to bypass the seals. Chen et al. [24] concluded that the AE RMS values were closely related to the internal leakage rate. Therefore, a similar correlation based on similar leakage modes provides further evidence that the leakage rate is closely related to the AE signal energy.

To estimate the RUL, a timeseries model of the RMS signal process must be created to be able to forecast the values. For the steadily increasing trend seen during the continuous operation of the test rig, a simple regression model may suffice. However, the model should also be able to predict the RMS values when the continuous operation is paused. The response seen in the RMS signal poses a challenge to the prediction model because the large drop in energy complicates the modelling process. However, for continuously operating cylinders, or well-scheduled operations, in which the drop in RMS value can be foreseen, these issues will not pose a problem, and a simpler model can possibly be applied.

## 5. Conclusions

This study investigated the relationship between AE features and the initiation of external fluid leakage due to seal wear in a hydraulic test rig. Experiments were conducted on a hydraulic test rig by varying the location of the sensor and the rod speed conditions for the RTF tests. The AE signal recorded from the experiments was analyzed using different time and frequency domain features. Important findings from the AE data analysis are as follows:

- The AE RMS feature was observed to be promising for direct application as a CM indicator to monitor external leakage in the hydraulic test rig. The intermittent sampling of data during accelerated leakage initiation testing showed steadily increasing RMS feature values until leakage was initiated. After the leakage was initiated, the RMS feature values continued to rise until settling at a steady level.
- The AE RMS feature showed a linear increasing trend with increasing leakage when the AE sensor was mounted on the flange section and bandpass filtered between 60 and 80 kHz.
- The AE RMS feature at leakage initiation was seen to be promising due to being scalable between different sensor locations and rod speed conditions. For the different RTF tests, the ratio between different sensor locations and rod speed conditions varied by 1.4037% and 1.4035%, respectively.
- The scaling factors applied showed excellent results in closely estimating a similar leakage threshold as the true threshold value.

Further work on creating forecasting models of the RMS signal should be conducted. Further RTF tests with similar conditions should also be conducted for repeatability. The research presented in this paper provides a strong foundation for future research on RUL estimation for predictive maintenance based on AE features for hydraulic rod seals.

## Figures and Tables

**Table 1:** Test rig details.

**Primary seal**	Stepseal V, Turcon^®^PTFE (Supplier: Trelleborg Sealing Solutions)
**Secondary seal**	Stepseal 2K, Turcon^®^PTFE (Supplier: Trelleborg Sealing Solutions)
**Excluder**	WE510050, Turcon^®^PTFE (Supplier: Trelleborg Sealing Solutions)
**Wiper**	Hallite 38, Polyester based TPE (Supplier: Hallite)
**Bearing strip**	GR6900500-C380, Turcite^®^(Supplier: Trelleborg Sealing Solutions)
**Rod material**	42CrMo4(+QT) with 20 µ HCr
**Hydraulic fluid**	Shell Tellus S2 VX 46
**AE sensor**	R15α (Supplier: Physical Acoustics)

**Table 2:** RTF test conditions.

Test No.	Pressure	Piston Rod Speed	Sensor Location	Run Time	Seal Conditions at Start of Test
**1**	15 bar	25 mm/s	rod	Until leakage	All seals unworn
**2**	15 bar	15 mm/s	flange	Until leakage	Main and secondary unworn, rest worn from previous test
**3**	15 bar	15 mm/s	rod	Until leakage	Main and secondary unworn, rest worn from previous test
**4**	15 bar	25 mm/s	flange	Until leakage	Main and secondary unworn, rest worn from previous test
**5**	15 bar	15 mm/s	flange	Five days or until total seal failure	Main and secondary unworn, rest worn from previous test

**Table 3:** Equations for the extracted features [19].

**RMS**	$x_{rms}=\frac{\sum_{n=1}^{N}x{\left(n\right)}^{2}}{N}$
**Peak**	$x_{peak}=\max\left|x\left(n\right)\right|$
**Skewness**	$x_{skew}=\frac{\sum_{n=1}^{N}{\left(x\left(n\right)-x_{m}\right)}^{3}}{\left(N-1\right)x_{std}^{3}}$
**Crest Factor**	$x_{crest}=\frac{x_{peak}}{x_{rms}}$
**Kurtosis**	$x_{kurt}=\frac{\sum_{n=1}^{N}{\left(x\left(n\right)-x_{m}\right)}^{4}}{\left(N-1\right)x_{std}^{4}}$
**Mean frequency**	$x_{meanf}=\frac{\sum_{k=1}^{K}f_{k}s\left(k\right)}{\sum_{k=1}^{K}s\left(k\right)}$
**Median frequency**	$x_{medf}=\frac{1}{2}\overset{K}{\underset{k=1}{\sum }}s\left(k\right)$

**Table 4:** Equations for calculating sensor location and rod speed scaling factors.

Location Scale	Speed Scale
$\frac{x_{rms4}\left(L_{4}\right)}{x_{rms1}\left(L_{1}\right)}$	$\frac{x_{rms1}\left(L_{1}\right)}{x_{rms3}\left(L_{3}\right)}$
$\frac{x_{rms2}\left(L_{2}\right)}{x_{rms3}\left(L_{3}\right)}$	$\frac{x_{rms4}\left(L_{4}\right)}{x_{rms2}\left(L_{2}\right)}$

**Table 5:** Calculated scaling factors between different sensor locations and rod speed conditions.

Location Scale 1	Location Scale 2	Speed Scale 1	Speed Scale 2
2.4791	2.5139	1.3537	1.3727

## Data Availability

The authors confirm that, data supporting the findings will be made available upon request.
